# Relationship Among Chlamydia and Mycoplasma Pneumoniae Seropositivity, *IKZF1* Genotype and Chronic Obstructive Pulmonary Disease in A General Japanese Population: The Nagahama Study

**DOI:** 10.1097/MD.0000000000003371

**Published:** 2016-04-18

**Authors:** Shigeo Muro, Yasuharu Tabara, Hisako Matsumoto, Kazuya Setoh, Takahisa Kawaguchi, Meiko Takahashi, Isao Ito, Yutaka Ito, Kimihiko Murase, Chikashi Terao, Shinji Kosugi, Ryo Yamada, Akihiro Sekine, Takeo Nakayama, Kazuo Chin, Michiaki Mishima, Fumihiko Matsuda

**Affiliations:** From the Department of Respiratory Medicine (SM, HM, II, YI, KM, MM); Center for Genomic Medicine, Kyoto University Graduate School of Medicine (YT, KS, TK, MT, CT, RY, FM); Department of Medical Ethics and Medical Genetics, Kyoto University School of Public Health, Kyoto (SK); Center for Preventive Medical Science, Chiba University, Chiba (AS); Department of Health Informatics, Kyoto University School of Public Health (TN); and Department of Respiratory Care and Sleep Control Medicine, Kyoto University Graduate School of Medicine, Kyoto, Japan (KC).

## Abstract

Chronic obstructive pulmonary disease (COPD) is a possible risk factor for cardiovascular disease. The association of COPD with the pathogenicity of infection with *Chlamydia pneumoniae* and *Mycoplasma pneumoniae* is controversial. We conducted a cross-sectional study to clarify the association between atypical *pneumoniae* seropositivity and COPD in a general population. We also investigated genetic polymorphisms conferring susceptibility to a *pneumonia* titer.

The study included 9040 Japanese subjects (54 ± 13 years). COPD was defined as a ratio of forced expiratory volume in 1 second to forced vital capacity of less than 70%. Serum levels of IgA and IgG antibodies to *C pneumoniae* were determined using an enzyme-linked immunoassay, and *M pneumoniae* seropositivity was assessed by a particle agglutination test.

Subjects seropositive for *C pneumoniae* (26.1%) had a higher prevalence of COPD (seropositive, 5.8%; seronegative, 3.1%; *P* < 0.001) after adjustment for age, sex, height, weight, and smoking status. The association between *M pneumoniae* seropositivity (20.4%) and COPD was also significant in covariate-adjusted analysis (*P* < 0.001). A genome-wide association analysis of the *C pneumoniae* IgA index identified a susceptible genotype (rs17634369) near the *IKZF1* gene, and the seropositive rate of *C pneumoniae* significantly differed among genotypes (AA, 22.5; AG, 25.3; GG, 29.7%, *P* < 0.001). On multiple regression analysis, seropositivity for both *C pneumoniae* (odds ratio = 1.41, *P* = 0.004) and *M pneumoniae* (odds ratio = 1.60, *P* = 0.002) was an independent determinant for COPD, while no direct association was found with the rs17634369 genotype.

Seropositivity for both *C pneumoniae* and *M pneumoniae* is an independent risk factor for COPD in the general population.

## INTRODUCTION

Chronic obstructive pulmonary disease (COPD), as assessed by forced expiratory volume in 1 second (FEV_1_) and forced vital capacity (FVC), is an independent risk factor for the incidence of cardiovascular diseases, including stroke,^1^ myocardial infarction,^2^ and heart failure,^3,4^ as well as for dementia^5^ in the general population. COPD has also been associated with increased mortality.^6^ Although the most important risk factor for COPD is long-term smoking,^7^ only a small proportion of smokers develop airflow obstruction,^8^ and some lifelong nonsmokers develop COPD.^9^ These inconsistent results suggest that other risk factors are also causative in the pathogenesis of COPD.

Factors postulated to play a role in the pathogenesis of COPD to date include air pollution, respiratory infection in childhood, bronchial hyperresponsiveness, systemic inflammation, and genetic background.^7,10,11^ Infection with *Chlamydia pneumoniae* (*C pneumoniae*), the most common nonviral human respiratory pathogen, has also been proposed as a risk factor for COPD.^12^ Although the mechanism by which chronic infection with *C pneumoniae* might trigger the development of COPD is not precisely understood, 1 plausible mechanism may be the release of inflammatory cytokines and subsequent airway inflammation and tissue damage.^12^ Higher titers of *C pneumoniae* in blood specimens are commonly observed in patients with respiratory diseases, namely COPD,^13^ chronic bronchitis,^14^ and symptomatic respiratory disease,^15^ and in hospitalized patients with acute exacerbation of COPD.^16^ Further, approximately 50% of cases of exacerbated COPD are caused by bacterial infection, such as with *C pneumoniae*.^17^ Although the prevalence and mortality of COPD are increasing worldwide,^18^ the involvement of *C pneumoniae* in the development of COPD in the general population is largely unknown. *Mycoplasma pneumoniae* (*M pneumoniae*) is another atypical pathogen that causes community-acquired pneumonia. Infection or colonization of *M pneumoniae* in the lower airway is associated with the pathogenesis of bronchial asthma,^19^ suggesting that chronic infection with *M pneumoniae* may also be a risk factor for COPD.

Recent advances have enabled the analysis of millions of single nucleotide polymorphisms (SNPs) dispersed throughout the human genome. Genome-wide association studies (GWAS) conducted without a prior hypothesis have successfully identified susceptibility loci for various common diseases and quantitative traits. GWAS of lung function have identified multiple loci such as the hedgehog interacting protein (*HHIP)* and glutathione S-transferase C-terminal domain containing (*GSTCD)* gene region.^20^ Despite the possible existence of SNPs which might influence susceptibility to seropositivity for *pneumoniae*, and the relationship between *C pneumoniae* and *M pneumoniae* infection and pulmonary function, no study has yet explored SNPs associated with seropositivity for both strains.

Here, we conducted a cross-sectional study to investigate the association of *C pneumoniae* and *M pneumoniae* seropositivity with pulmonary function in a large general Japanese population. We also conducted a GWAS to explore SNPs for their association with seropositivity for both strains.

## METHODS

### Study Subjects

Study subjects were participants in the Nagahama Prospective Genome Cohort for Comprehensive Human Bioscience (the Nagahama Study). The Nagahama Study cohort was recruited between 2008 and 2010 from the general population (30–74 years old) living in Nagahama City, a largely suburban city of 125,000 inhabitants in Shiga Prefecture. Nagahama City residents aged 30 to 74 years at recruitment and without serious health problems who agreed to participate in the cohort study of their own accord were recruited via mass communications in the local community, such as public relations magazines and periodical newspapers.

Among a total of 9804 participants, we considered 9237 subjects as the total population in this study, consisting of 3246 subjects whose genome-wide SNP genotype data were available and 5991 other subjects as a subset of the remaining samples available for replication genotyping (Table S1). Among these, participants meeting any of the following conditions were excluded from subsequent association analysis for COPD: pregnancy (n = 40), history of lung cancer (n = 19), unsuccessful assessment of the Brinkman index (n = 23) or spirometric parameters (n = 24), and unavailability of serum *M pneumoniae* titer (n = 1) or rs17634369 genotype (n = 90). Eventually, a total of 9040 people were included as study subjects.

All study procedures were approved by the ethics committee of Kyoto University Graduate School of Medicine and the Nagahama Municipal Review Board. Written informed consent was obtained from all participants.

### Basic Clinical Parameters

Clinical measurements and blood sampling were performed at enrollment. Medical history and smoking status were investigated using a structured questionnaire. The Brinkman index was calculated as the daily number of cigarettes smoked multiplied by the number of years spent smoking.

### Evaluation of Pulmonary Function

Pulmonary function was measured by an FVC maneuver on a computed spirometer with automated quality checks (SP-350 COPD, Fukuda Denshi, Tokyo, Japan). Prebronchodilator spirometry was measured by certified medical technologists in accordance with a standardized protocol. COPD was defined by a ratio of FEV_1_ to FVC of less than 70%. Predicted normal values for FVC (FVC predicted) (L) and FEV1 (FEV_1_ predicted) (L) were calculated using the following equations, in accordance with guidelines developed by the Japanese Respiratory Society for the diagnosis and treatment of COPD (3rd edition; http://www.jrs.or.jp/uploads/uploads/files/photos/765.pdf): FVC male = 0.042 × height (cm) − 0.024 × age − 1.785; FVC female = 0.031 × height − 0.019 × age − 1.105; FEV_1_ male = 0.036 × height − 0.028 × age − 1.178; FEV_1_ female = 0.022 × height − 0.022 × age − 0.005.

### Measurement of *C pneumoniae* IgA and IgG Indices

Levels of serum IgA and IgG antibodies to *C pneumoniae* were determined using a specific enzyme-linked immunoassay (ELISA) kit (HITAZYME *C pneumoniae*, Hitachi Chemical, Tokyo, Japan) that detects antibodies to the chlamydial outer membrane complex. IgA and IgG levels in each sample were expressed as the IgA or IgG index, respectively. Seropositivity to *C pneumoniae* was diagnosed as an IgA and IgG index value of more than 1.1 for both. Details of the ELISA assay and validity of the cut-off point have been described elsewhere.^21^ Mean intraassay coefficients of variation were 3.3% to 7.7% for the IgA index and 6.3% to 9.0% for the IgG index. Respective mean interassay coefficients of variation were 3.8% to 10.7% and 4.0% to 10.7%.

### Evaluation of *M pneumoniae* Seropositivity

*M pneumoniae* seropositivity was assessed using a semiquantitative particle agglutination test kit (Serodia-Myco II, Fujirebio, Tokyo, Japan) consisting of gelatin particles coated with cell membrane components of *M pneumoniae* (Mac strain).^22^ Serum samples were serially diluted to give final dilutions of 1:40 to 1:20, 480. According to the manufacturer's instructions, antibody titers of 1:40 and higher were considered seropositive.^23,24^

### Genome-Wide SNP Genotyping

DNA was extracted from peripheral blood samples by the phenol–chloroform method. Genome-wide SNP genotyping was performed on 3710 samples of participants who joined the Nagahama cohort from 2008 to 2009. A series of BeadChip DNA arrays were used for analysis, namely HumanHap610 quad (1828 samples), HumanOmni2.5-4 (1616 samples), HumanOmni2.5-8 (378 samples), HumanOmni2.5s (192 samples), and HumanExome (192 samples) (Illumina, San Diego, CA). Several samples were repeatedly genotyped using different arrays. As a reference panel, 192 samples were genotyped using HumanOmni2.5-8, HumanOmni2.5s, and HumanExome, and used in the following genotype imputation. Genotyping quality was controlled by excluding SNPs with a call rate below 99%, minor allele frequency below 0.01, or extreme deviation from Hardy–Weinberg equilibrium (*P* < 1.0 × 10^−7^). This threshold was defined to ensure a concordance ratio of SNPs genotyped by different SNP arrays greater than 99.99%.^25^ Genotype imputation was performed by a standard 2-step procedure using MACH ver. 1.0.16 software with 1792015 SNPs commonly genotyped in the 192 samples as a reference. Imputed SNPs with a minor allele frequency of less than 0.01 or *R*^2^ less than 0.5 were excluded from the following association analysis. Samples with a call rate less than 95% (n = 162) were excluded from analysis. Of the remaining 3548 subjects, 295 individuals were excluded as they showed high degrees of kinship (Pi-hat greater than 0.35, PLINK ver. 1.07), and 7 individuals were excluded as ancestry outliers, as identified by principal component analysis using the HapMap Phase 2 release 28 JPT dataset as reference (EIGENSTRAT ver. 2.0).

### Replication Genotyping

Replication analysis was performed with a subset of the remaining samples from the Nagahama cohort (n = 5991). Genotypes were analyzed with a TaqMan probe assay using commercially available primer and probe sets purchased from Life Technologies Corporation (Carlsbad, CA). Fluorescence level of PCR products was measured using the 7900HT Fast Real-Time PCR System (Life Technologies).

### Statistical Analysis

Genome-wide association analysis of the *C pneumoniae* IgA and IgG index values was performed by linear regression analysis under an additive genetic model adjusted for age, age-squared, sex, and body mass index, while analysis of *M pneumoniae* seropositivity was performed by a Chi-square test (PLINK). Rank-based inverse normal transformation was applied to the *C pneumoniae* IgA and IgG index values. Population stratification was adjusted using top principal components as covariates. Given an estimation of the appropriate testing burden in a GWAS in Europeans as a million, a *P*-value less than 5.0 × 10^−8^ was considered to have genome-wide significance.^26^ Based on findings that an additive model has reasonable power in detecting both additive and dominant effects,^27^ we applied only an additive model in the current GWAS.

In other association analyses, differences in numeric variables among subgroups were assessed by analysis of variance, while differences in frequency were assessed by a Chi-square test. Factors independently associated with COPD were identified by multiple logistic regression analysis. All statistical analyses were performed using JMP 9.0.2 software (SAS Institute, Cary, NC), with a conventional *P*-value less than 0.05 considered to indicate statistical significance.

## RESULTS

Table 1 shows the clinical characteristics of the study subjects. A total of 341 (3.8%) subjects were diagnosed with COPD. Subjects with COPD were significantly older (COPD 61 ± 12 vs control 53 ± 13 years, *P* < 0.001), and mostly male (61.0% vs 31.6%, *P* < 0.001) and current or past smokers (58.7% vs 33.8%, *P* < 0.001). Differences in major clinical parameters by severity of pulmonary function are detailed in Table S2.

**TABLE 1:**
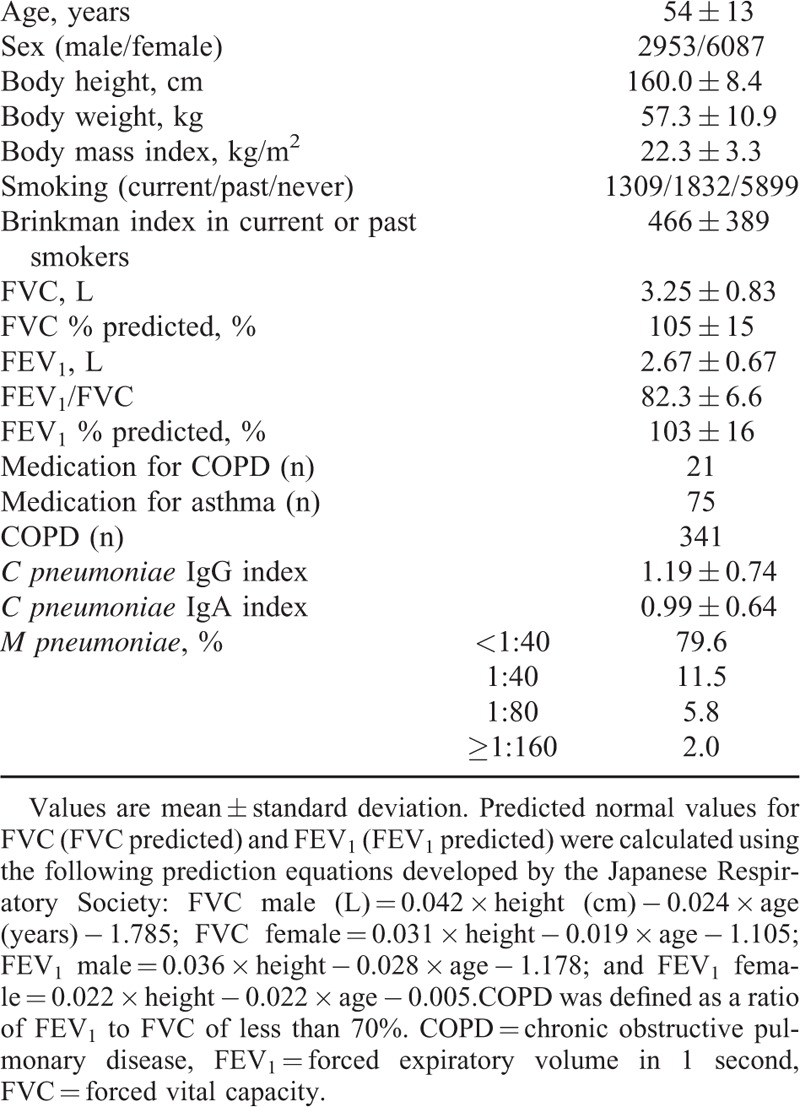
Clinical Characteristics of Study Subjects (n = 9040)

### *C pneumoniae* and *M pneumoniae* Seropositivity With Pulmonary Function

**FIGURE 1:**
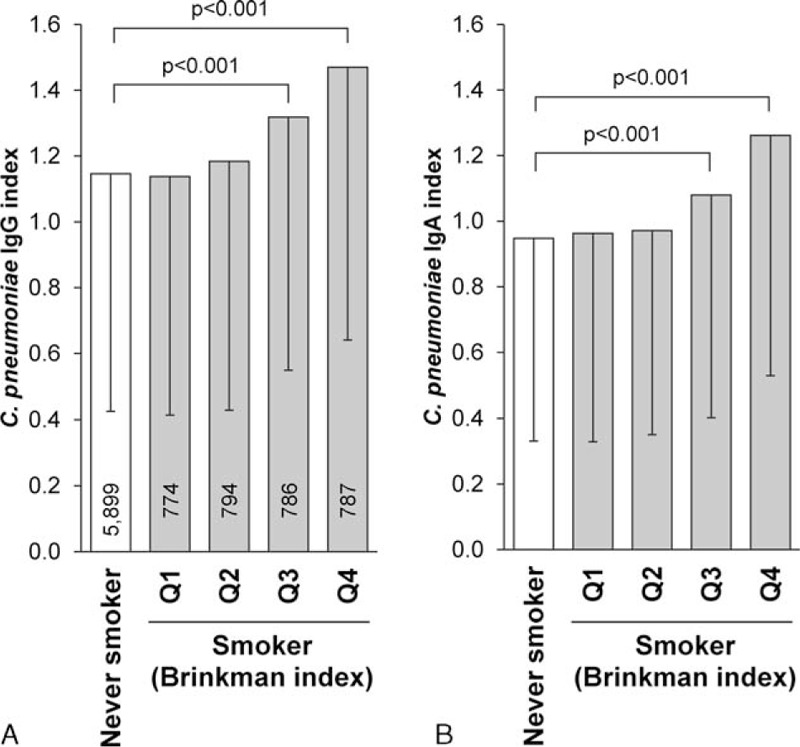
Mean IgA and IgG indices by smoking status and quartile of Brinkman index. Values are mean ± standard deviation of IgG (A) and IgA (B) indices. Smokers were divided into quartiles according to Brinkman index (Q1: <159.7, Q2: 160.0–374.0, Q3: 375.0–684.8, Q4: 685.0–2784). Numbers of subjects in each subgroup are shown in the column. Statistical significance was assessed by analysis of covariance and post-hoc analysis was performed by Dunnett test.

The frequency of *C pneumoniae* and *M pneumoniae* seropositivity was 26.1% and 20.4%, respectively. The number of subjects seropositive for *C pneumoniae* increased with age, whereas that for *M pneumoniae* showed the opposite relationship (Figure S1). Associations of seropositivity for both strains with pulmonary function are summarized in Table 2 and Figure S2. Subjects seropositive for *C pneumoniae* exhibited significantly lower pulmonary function and a higher frequency of COPD. Although the values of the *C pneumonia* e IgA and IgG indices were linearly associated with smoking intensity as assessed by the Brinkman index (Figure 1), significantly lower pulmonary function in seropositive subjects remained after adjustment for the interrelationships (Table 2). Conversely, the pulmonary function of subjects seropositive for *M pneumoniae* was slightly better than that of seronegative subjects (Table 2), but these associations were lost after adjustment for covariates, including age, presumably due to the markedly younger age of seropositive subjects. In contrast, the association between *M pneumoniae* seropositivity and COPD became significant in the covariate-adjusted analysis (odds ratio = 1.63). Similar results were observed in an analysis which excluded subjects receiving treatment for asthma or COPD (n = 90) (Table S3).

**TABLE 2:**
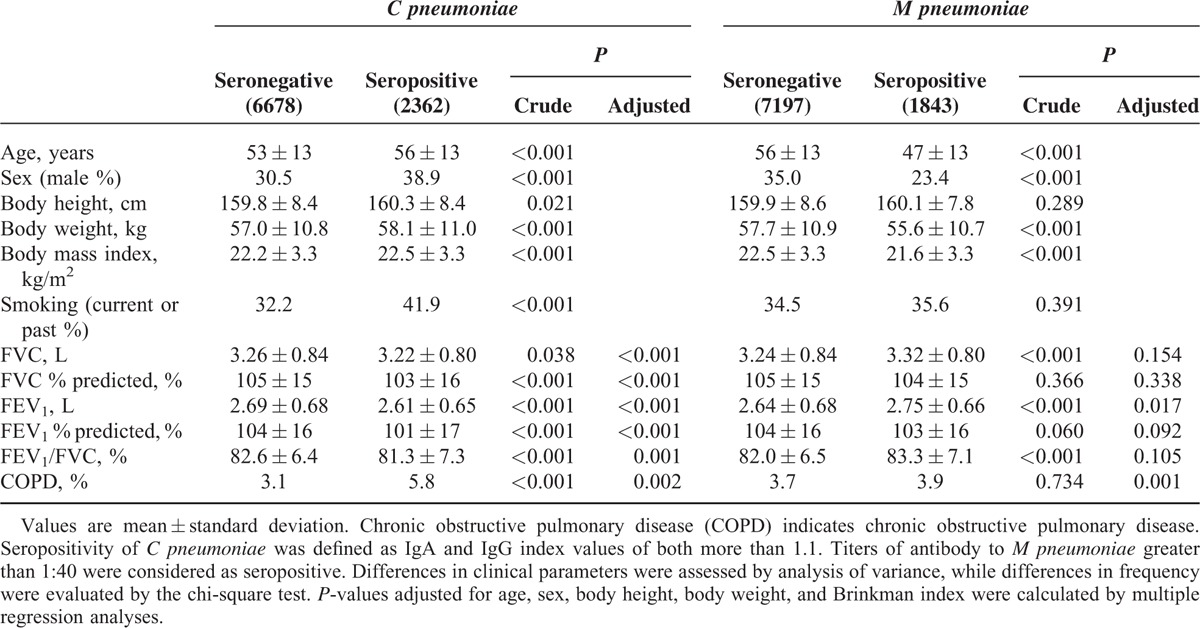
Differences in Clinical Parameters of *C pneumoniae* and *M pneumoniae* Seropositive Patients

### GWAS of *C pneumoniae* IgA and IgG Indices

GWAS of the *C pneumoniae* IgA index values identified strong association signals at 6p22.3 (rs9460391, β = 0.163, *P* = 6.9 × 10^−8^), 7p12 (rs17634369, β = 0.134, *P* = 3.0 × 10^−8^), and 13q33 (rs942102, β = −0.134, *P* = 7.7 × 10^−8^) (Figure S3, Table S4). Additional genome-wide significant signals were found in the human leukocyte antigen gene region, but these were lost after adjustment for population stratification (Figures S4 and S5). No significant signals were found in GWAS of the *C pneumoniae* IgG index value (Figures S3 and S4), or in analysis of *M pneumoniae* seropositivity (Figure S6). Statistical power of the GWAS is shown in Figure S8. Additional susceptible SNPs with a lower frequency or smaller effect size might be detectable with a greater number of subjects.

### Replication Analysis for the Candidate SNPs

Replication analysis of the remaining Nagahama samples confirmed the positive association of rs17634369 (β = 0.080, *P* = 1.4 × 10^−5^), but not rs9460391 (*P* = 0.414) or rs942102 (*P* = 0.621) (Table S4). Per-allele effect size of rs17634369 on the *C pneumoniae* IgA index value calculated from the combined datasets used in the GWAS, and replication analysis was approximately 0.1 (*P* = 1.3 × 10^−11^). SNP rs17634369 was located approximately 22 kb upstream of the *IKZF1* gene, where no other genes were mapped (Figure S7), and was in strong linkage disequilibrium with the SNP rs4917014 (D′ = 0.921, *r*^2^ = 0.773), which was previously identified as conferring susceptibility to systemic lupus erythematosus (SLE).^28^ There were no significant associations between previously validated SNPs for SLE and *C pneumoniae* IgA index value (Table S5).

### SNP rs17634369 and *C pneumoniae* IgA Index

**FIGURE 2:**
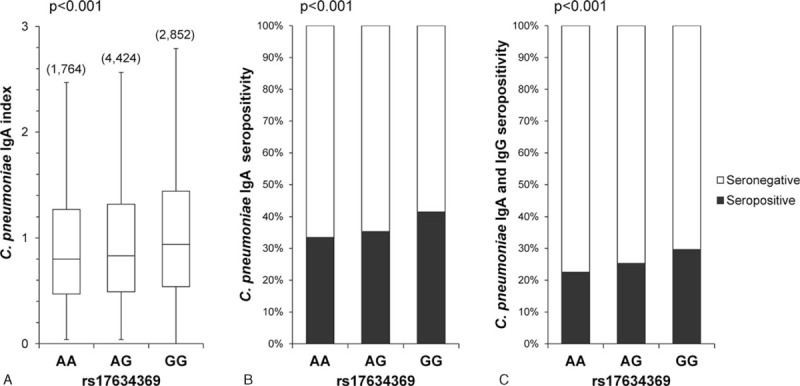
Association of rs17634369 genotype with *C pneumoniae* IgA index value and seropositivity. (A) Box plot of *C pneumonia* e IgA index by rs17634369 genotype. Number of subjects for each genotype is shown in parentheses. Statistical significance was assessed by analysis of variance. (B) Seropositivity of *C pneumoniae* defined by the IgA index. An IgA index value of more than 1.1 was considered as seropositive. Statistical significance was assessed by the Chi-square test. (C) Seropositivity of *C pneumonia* e defined by the IgA or IgG index. Seropositivity was defined as IgA and IgG index values of more than 1.1 for both.

Figure 2A shows the differences in the *C pneumoniae* IgA index values for the rs17634369 genotype. Subjects with the G allele showed a significantly higher IgA index value. The seropositive rate of *C pneumoniae* defined by the IgA index (Figure 2B) and the IgA and IgG indices (Figure 2C) linearly increased with the number of G alleles. Associations between the rs17634369 genotype and *C pneumoniae* IgA index value (*P* < 0.001), as well as the seropositive rate defined by IgA and IgG indices (*P* < 0.001), remained significant after adjustment for age, sex, and smoking status.

### Multivariate Analysis for COPD

A multiple logistic regression analysis was used to further identify factors independently associated with COPD (Table 3). Results showed that both *C pneumoniae* and *M pneumoniae* seropositivity were independently associated with COPD (Model 1), even when individuals taking medication for asthma and COPD were excluded (Table S6), while the rs17634369 genotype was not directly associated with COPD (Model 1), even following adjustment for *C pneumoniae* and *M pneumoniae* seropositivity (Model 2). In a separate analysis by smoking status, positive associations between *pneumoniae* seropositivity and COPD were observed only in current or past smokers (Model 3), and not in never-smokers (Model 4). No interaction was observed between the rs17634369 genotype and *C pneumoniae* seropositivity (*P* = 0.846).

**TABLE 3:**
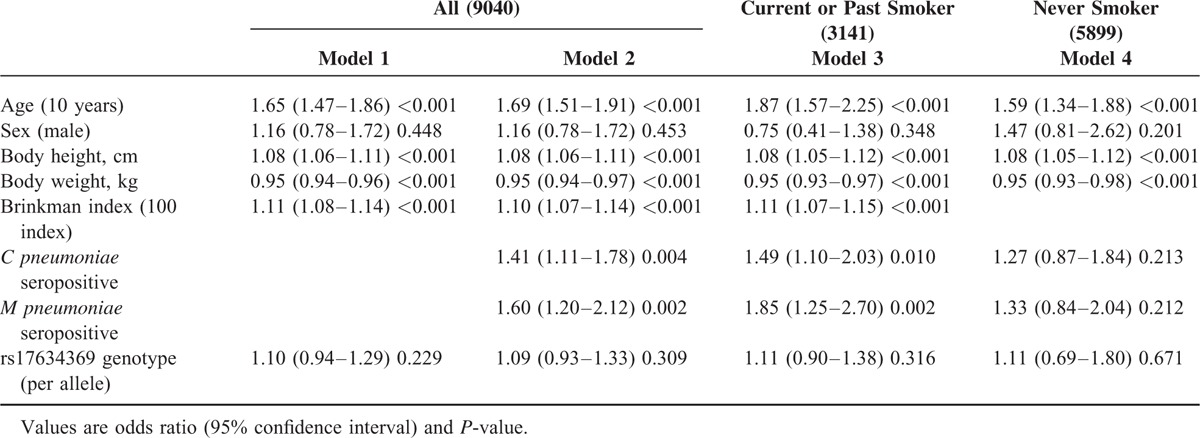
Multiple Logistic Regression Analysis for Chronic Obstructive Pulmonary Disease (COPD)

## DISCUSSION

In this study, we demonstrated that seropositivity for *C pneumoniae* and *M pneumoniae* was positively associated with COPD. To our knowledge, this is the 1st study to clearly show the risk of *C pneumoniae* and *M pneumoniae* seropositivity for COPD in a general population, particularly in smokers. Further, we identified SNPs conferring susceptibility for *C pneumoniae* seropositivity, although no direct association with COPD was observed.

Although several studies reported that patients with COPD had a significantly higher titer of serum *C pneumoniae* IgA or IgG,^13–15^ the East London COPD study in 110 patients with stable COPD reported an inconsistent result.^29^ A plausible reason for this discrepancy is insufficient statistical power due to the relatively small sample size. For example, von Hertzen et al^13^ compared IgA and IgG titers in 54 COPD patients and 321 healthy controls, and Brandén et al^15^ investigated the association of chronic *C pneumoniae* infection with longstanding airway symptoms in 199 patients, of whom 30 were diagnosed with COPD. In a general population, 1 study investigated the association of *C pneumoniae* infection with pulmonary function in 1773 men^30^ and failed to find any association with outcome measures, including baseline spirometric parameters and FEV_1_ decline during the 5-year follow-up period. However, the frequency of current or past smokers in the previous study (>80%) was more than double that in our present study, and more than double that in the National Survey on Circulatory Disorders of Japan,^31^ which reported that the frequency of current or past smokers in Japan is approximately 40%. The harmful effects of smoking might therefore render the potential pathogenicity of *C pneumoniae* infection undetectable. Given that our present study was based on a large population with a ratio of smokers to nonsmokers equivalent to that of the national survey, our observations may provide strong evidence that *C pneumoniae* seropositivity is a risk factor for reduced pulmonary function in general populations.

*M pneumoniae* is another atypical pathogen of community-acquired pneumonia. Although several small studies reported a possible risk of serologically identified *M pneumoniae* for acute exacerbation of COPD,^32,33^ other studies denied this etiological relationship.^34,35^ Clinical and epidemiological data on the pulmonary risk of *M pneumoniae* infection is thus limited and conflicting even in COPD patients. In addition, no data are available on the association between *M pneumoniae* infection and spirometric parameters. Our present findings are therefore important in helping to elucidate the pulmonary risk of *M pneumoniae* infection in a general population. Further, the risk of *M pneumoniae* infection was equal to or somewhat greater than that of *C pneumoniae*. Of note, however, subjects infected with *M pneumoniae* first appeared to have better pulmonary function, presumably due to their younger age.

In general populations, even mild to moderate airflow limitation and COPD have been suggested to confer a risk for the incidence of cardiovascular diseases.^2–5^ One potential factor in explaining the possible relationship between COPD and cardiovascular outcomes is atherosclerosis.^36^ Given that the present and previous reports showed a positive association between *C pneumoniae* seropositivity and atherosclerotic vascular change,^37,38^ as well as cardiovascular and all-cause mortality,^39^*C pneumoniae* infection might be a factor underlying the relationship between COPD and cardiovascular outcomes.

The rs17634369 genotype was significantly associated with the seropositive prevalence of *C pneumoniae*. According to the Encyclopedia of DNA Elements (ENCODE) project datasets (UCSC genome browser: http://genome.ucsc.edu/), this polymorphism lies approximately 22 kb upstream of the *IKZF1* gene. A recent systemic expression quantitative trait locus (eQTL) analysis^40^ found that the T allele of the rs4917014 genotype, a proxy of the G allele of the rs17634369 genotype, was associated with increased expression of the *IKZF1* gene (*cis*-effect). Further, very recently, the rs4917014 genotype was identified as susceptible for cold medicine-related Stevens–Johnson syndrome via a mechanism involving a change in the quantitative ratio of the *IKZF1* alternative splicing isoforms Ik1 and Ik2.^41^ Although we did not perform functional analysis for the relationship between rs17634369 or rs4917014 genotype and *C pneumoniae* Ig A index, these previous results are sufficient to consider *IKZF1* as a factor in *C pneumoniae* seropositivity.

*IKZF1* encodes the transcription factor “Ikaros.” Ikaros was reported to be associated with transcriptional regulation of human STAT4,^42^ which is involved in cell-mediated immune responses via Th1 cell development.^43^ The STAT4 pathway may therefore help explain the association between *IKZF1* genotype and *C pneumoniae* seropositivity. The eQTL analysis also reported a significant association between the rs4917014 genotype and increased expression of genes involved in the type 1 interferon response (*trans*-effect).^40^ Given that increased expression of interferon-α response genes is a distinct expression pattern in SLE patients,^44^ and that the rs4917014 genotype confers susceptibility to SLE,^28^ the increased *C pneumoniae* IgA levels and higher risk of SLE in individuals with the risk allele might partially share a common pathophysiological pathway.

The microimmunofluorescence (MIF) test is currently the objective standard for the serodiagnosis of *C pneumoniae*, but interlaboratory variability is an unanswered issue. An ELISA is another convenient method of serodiagnosis. However, discrepancies in the detection ratio between indirect (MIF, ELISA) and direct (PCR) methods remain to be resolved. Our present findings may suggest that the discrepancy in detection ratio among populations, which plays at least some role in the controversy over *C pneumoniae* infection in chronic inflammatory diseases, may in part be due to genetic heritability.^45^

Several limitations of our study warrant mention. First, we could not distinguish between persistent and past infection of *pneumoniae* by measuring seropositivity. Although the presence of *pneumoniae* antibodies may reflect the presence of prior infection, this may not be a valid measure of persistent and chronic active reinfection. A guideline from a workshop on the standardization of *C pneumoniae* diagnostic methods^46^ suggested that no valid serological marker specific for chronic *C pneumoniae* infection is available and that any interpretation of infection status on the basis of single titer readings should be done with care. Second, we might not have entirely excluded subjects with bronchial asthma due to a lack of postbronchodilator spirometry values, or subjects with other pulmonary diseases such as diffuse bronchiectasis and bronchiolitis obliterans. Given the positive association between atypical bacterial infection and asthma,^47^ our findings might have been confounded by interaction with the reversible airflow obstruction. However, our observed positive association between *C pneumoniae* and *M pneumoniae* seropositivity and COPD remained significant after the exclusion of subjects with self-reported medication for bronchial asthma or COPD, or with pulmonary disease. Third, as our study subjects were Japanese, the results of this study might not be simply extrapolatable to other populations with different environmental backgrounds. Further, the dbSNP database (http://www.ncbi.nlm.nih.gov/projects/SNP/) shows ethnic differences in the rs17634369 genotype, with the highest frequency of the G allele in Africans, followed by Asians and Caucasians.

In summary, our study clarified that dual *C pneumoniae* and *M pneumoniae* seropositivity is an independent risk factor for pulmonary function. Further investigations are needed to clarify mechanisms regarding the disease pathogenicity of *pneumoniae* infection.

## Supplementary Material

Supplemental Digital Content
