# Supplementary material for: Relationship Among Chlamydia and Mycoplasma Pneumoniae Seropositivity, IKZF1 Genotype and Chronic Obstructive Pulmonary Disease in A General Japanese Population: The Nagahama Study
Source: Medicine (Baltimore). 2016 Apr 18;95(15):e3371. doi: 10.1097/MD.0000000000003371 (PMC4839845; doi:10.1097/MD.0000000000003371)
Supplement: Supplemental Digital Content [file medi-95-e3371-s001.pdf]

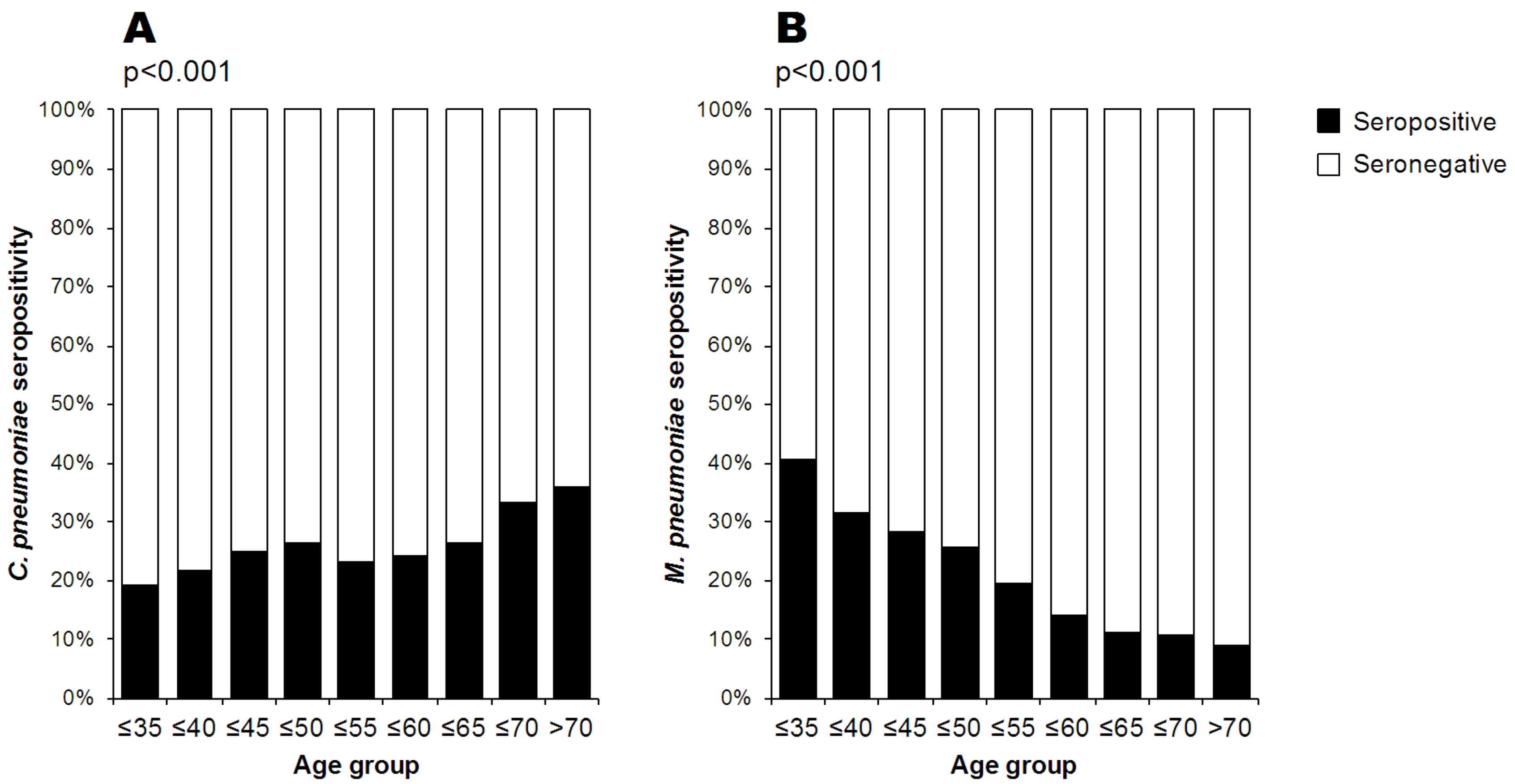

Supplementary Figure 1. *C. pneumoniae* and *M. pneumoniae* seropositivity by age group

A: *C. pneumoniae*, **B**: *M. pneumoniae*.

Seropositivity for *C. pneumoniae* was defined as both IgA and IgG index values of more than 1.1. Titers of antibody to *M. pneumoniae* greater than 1:40 were considered as seropositive. *P*-values adjusted for age, sex, body height, body weight, and smoking habit were calculated by multiple regression analyses.

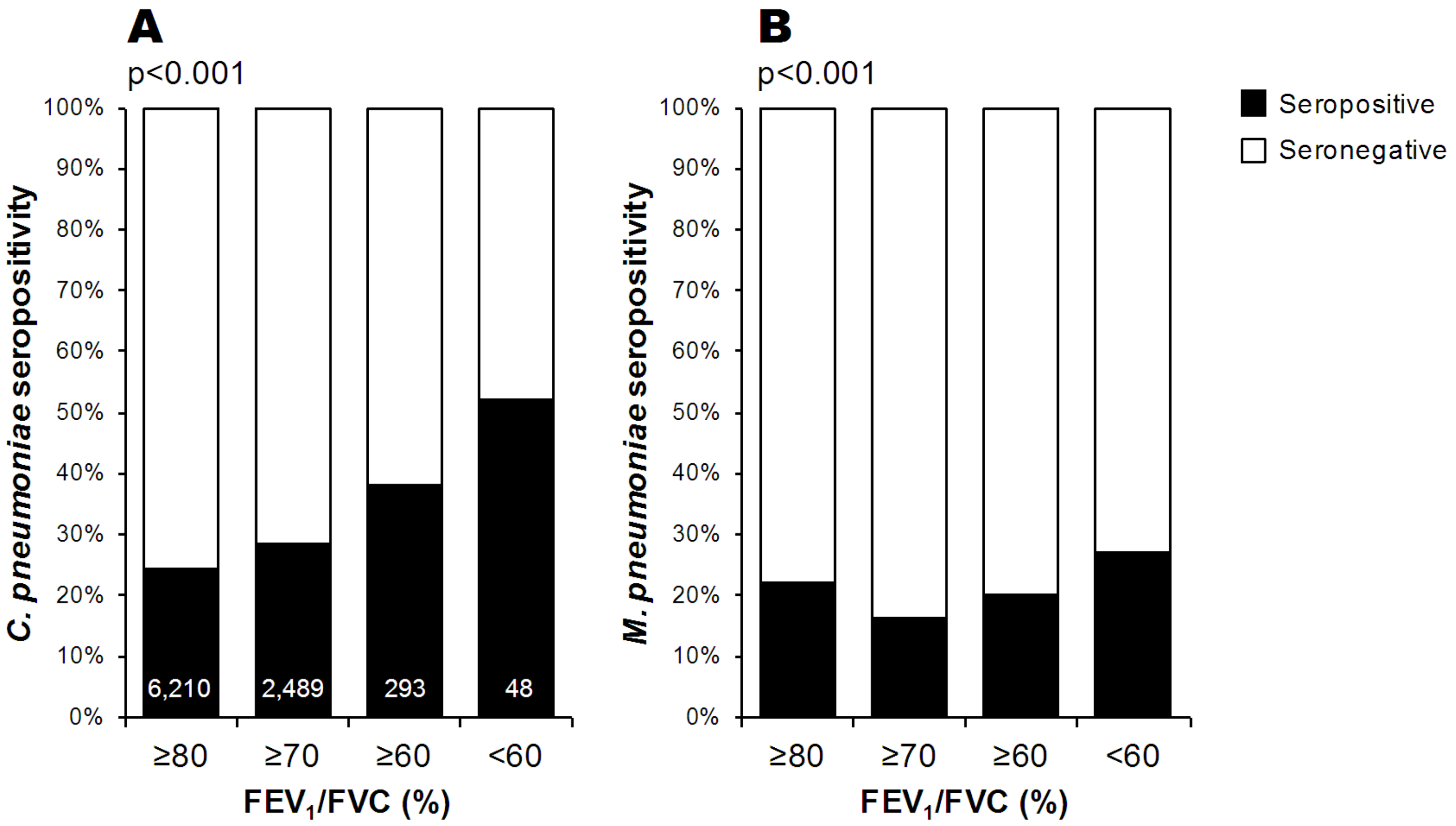

Supplementary Figure 2. *C. pneumoniae* and *M. pneumoniae* seropositivity by pulmonary function.

A: *C. pneumoniae*, **B**: *M. pneumoniae*.

Seropositivity for *C. pneumoniae* was defined as both IgA and IgG index values of more than 1.1. Titers of antibody to *M. pneumoniae* greater than 1:40 were considered as seropositive. Frequency difference was assessed by a chi-squared test.

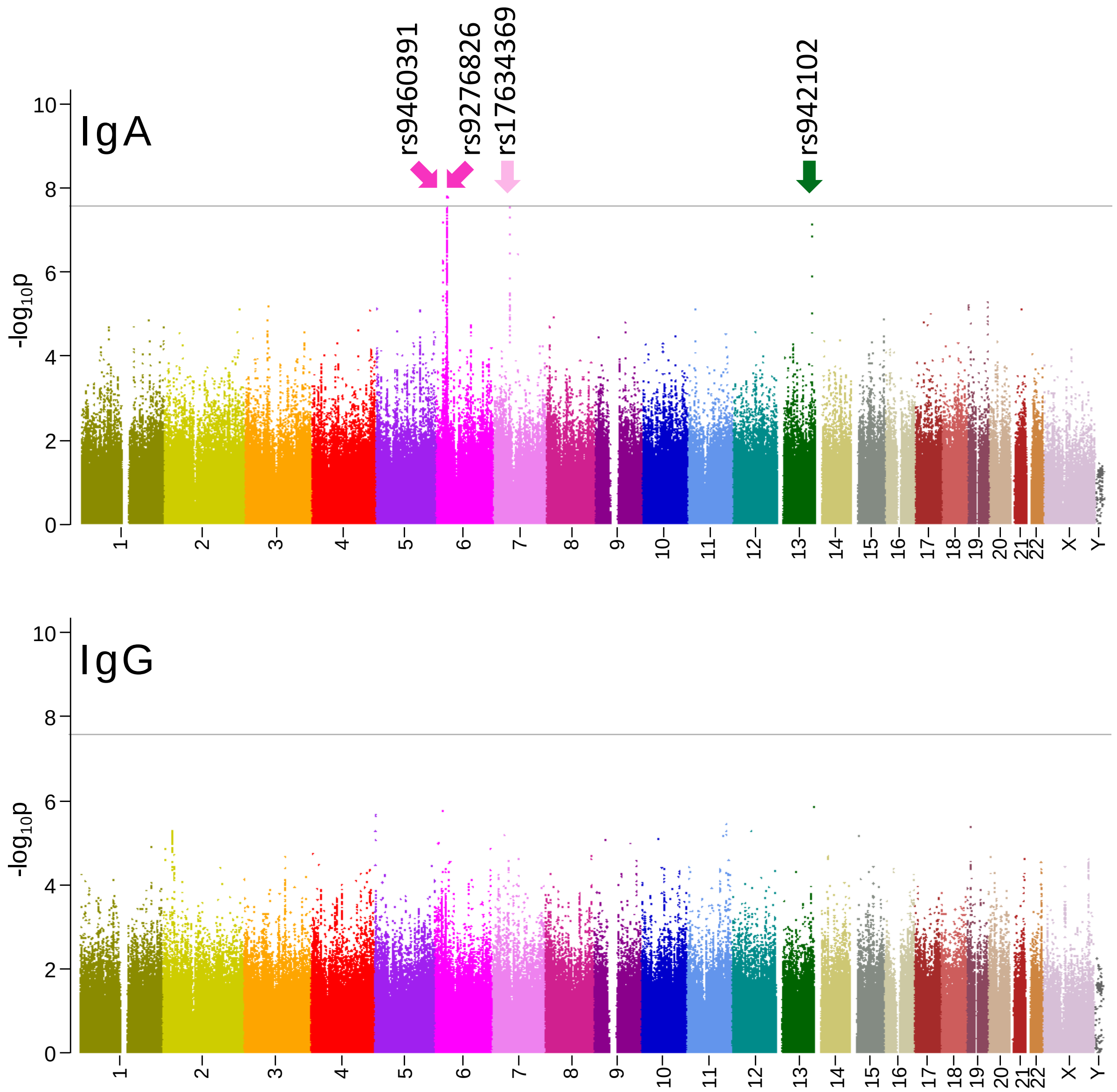

Supplementary Figure 3. Manhattan plots of population stratification-unadjusted association analysis of *C. pneumoniae* IgA and IgG indices

Rank-based inverse normal transformation was applied to serum values of the *C. pneumoniae* IgA and IgG indices to normalize their distributions. Adjusted factors in the association analysis were age, age squared, sex, and BMI. Association analysis was performed using PLINK v1.07 software.

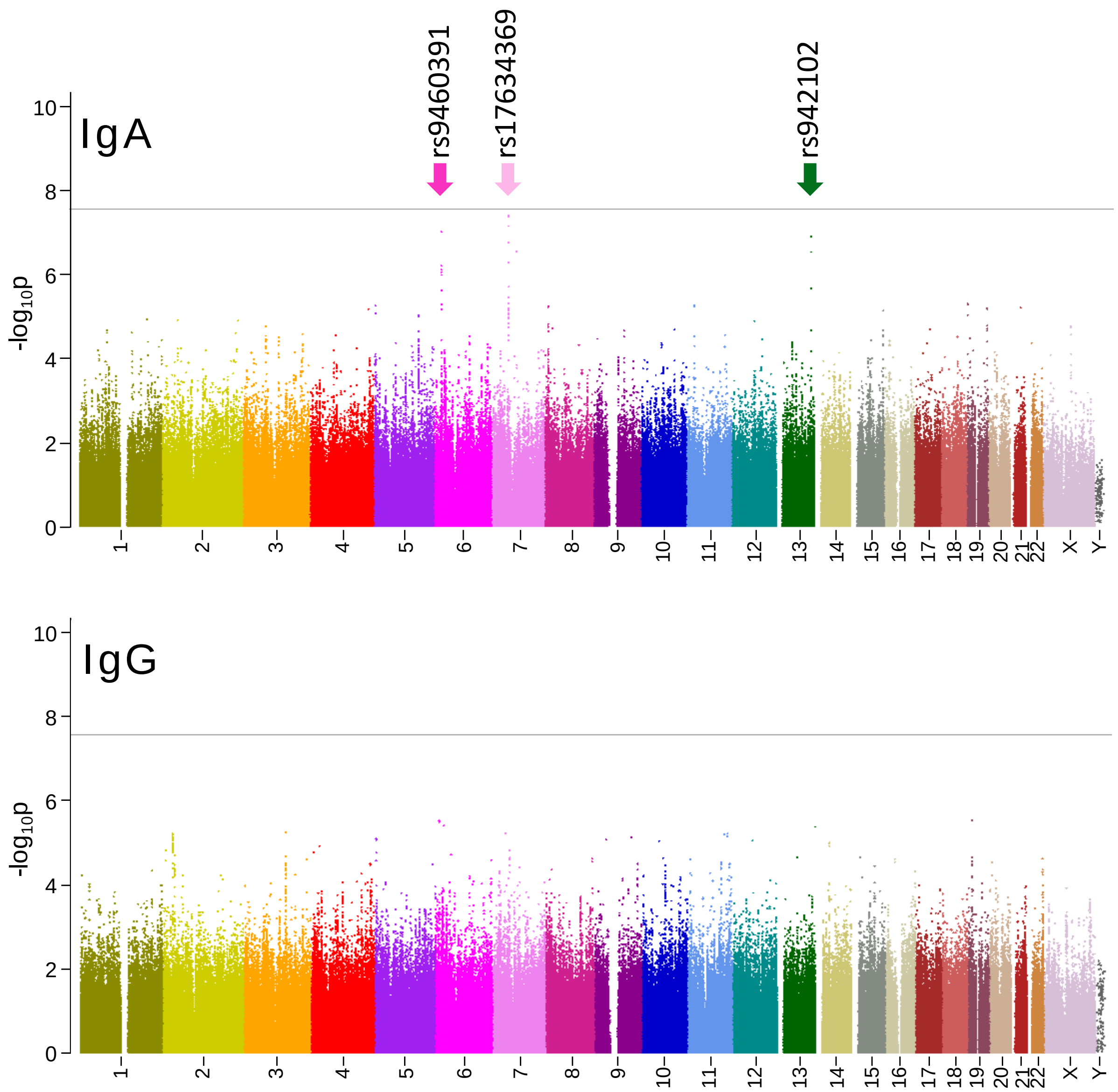

Supplementary Figure 4. Manhattan plots of population stratification-adjusted association analysis for *C. pneumoniae* IgA and IgG indices

Rank-based inverse normal transformation was applied to serum the values of the *C. pneumoniae* IgA and IgG indices to normalize their distributions. Adjusted factors in the association analysis were age, age squared, sex, and BMI. Population stratifications were corrected by principal components analysis[11]. Association analysis was performed using PLINK v1.07 software.

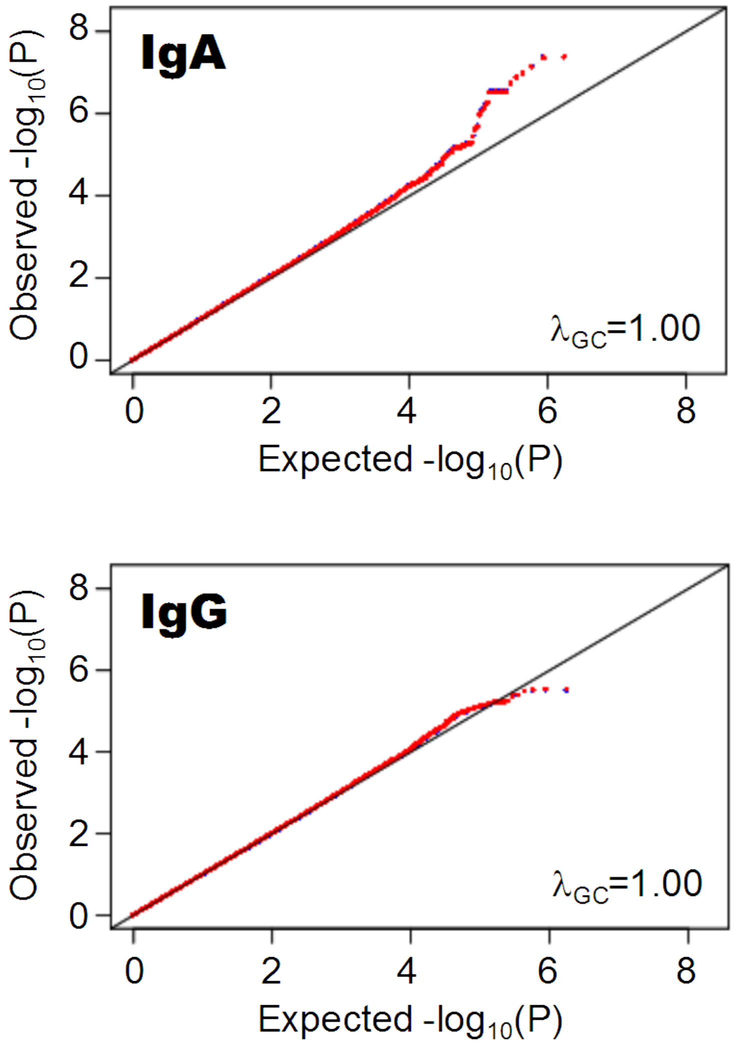

Supplementary Figure 5. Quantile-Quantile plots of the population stratification-adjusted association analysis for *C. pneumoniae* IgA and IgG indices

The Quantile-Quantile plot (QQ-plot) for the *P*-values of all SNPs that passed quality control criteria and for the *P*-values corrected for genomic control factor are indicated by blue and red dots, respectively. Lambda values before genomic control correction (λ_{GC}) are shown in each graph.

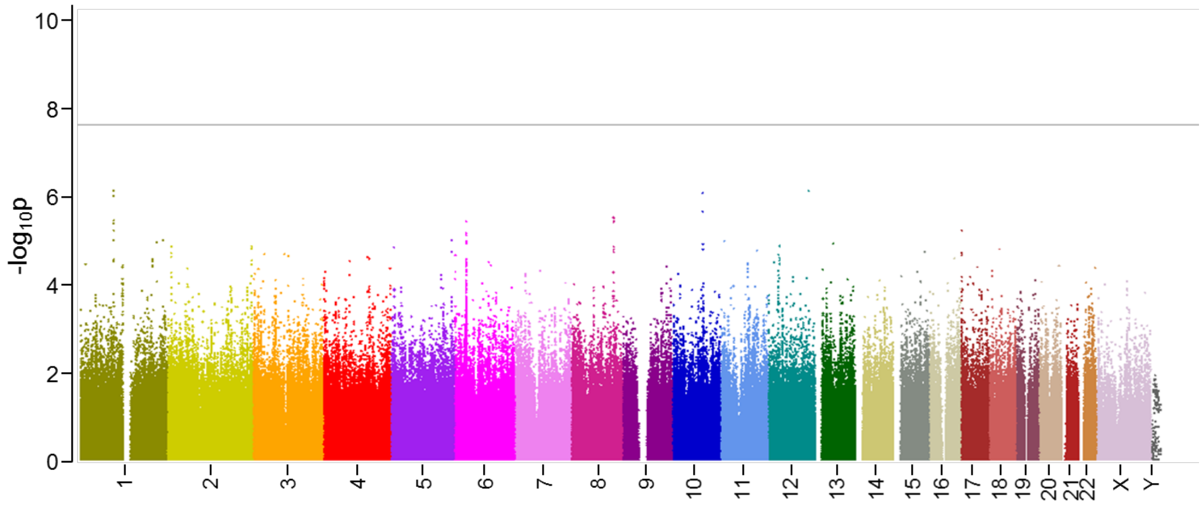

Supplementary Figure 6. Manhattan plots of population stratification-unadjusted association analysis for *M. pneumoniae* seropositivity

Titers of more than 1:40 were considered as seropositive. Association analysis was performed using PLINK v1.07 software.

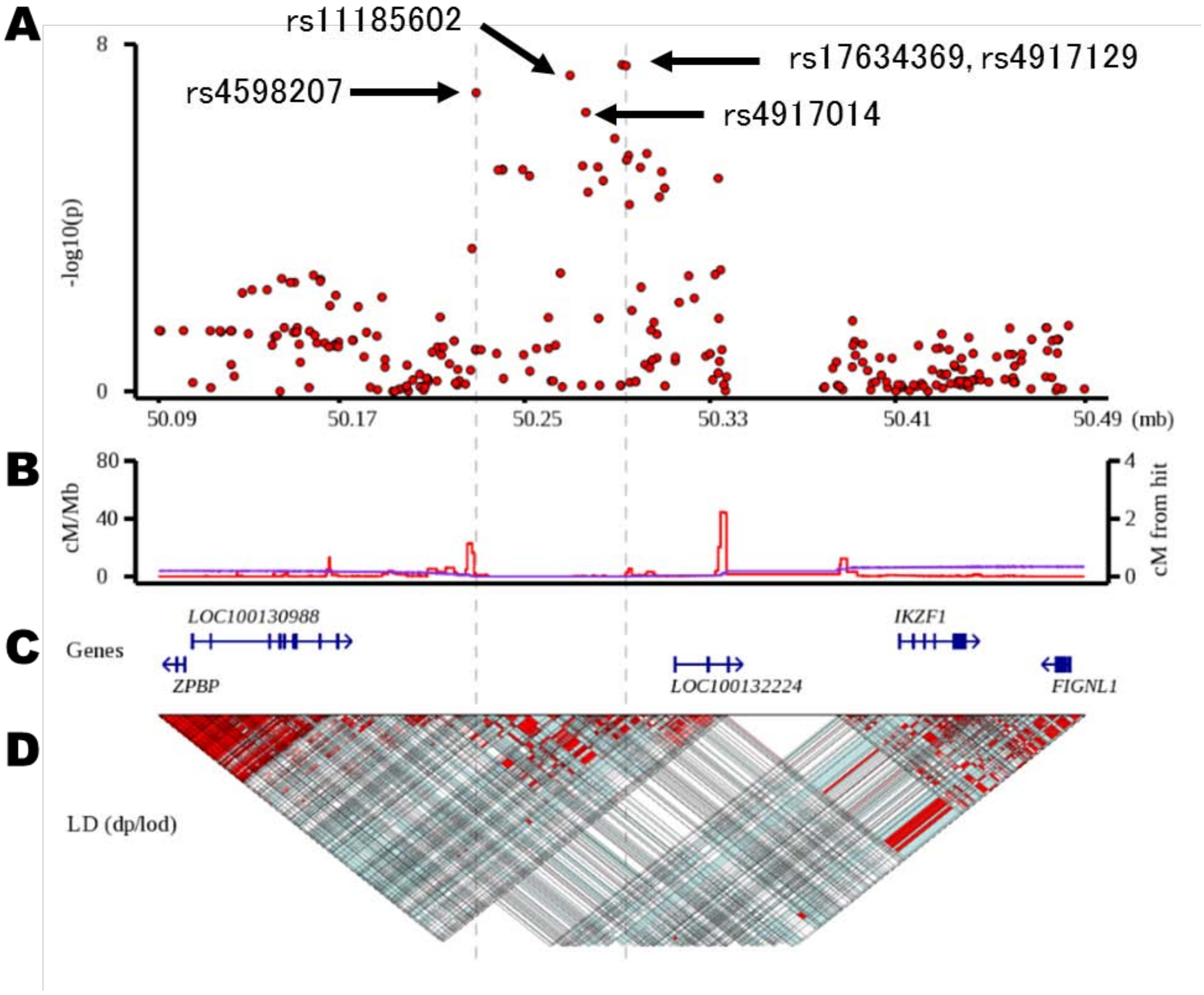

Supplementary Figure 7. Regional association plots of loci around the SNP rs17634369

A: *P*-values (-$\log_{10}$(p)) of genotyped SNPs around 200 kb to either side of the most strongly associated signal are shown in red dots. Genomic positions are based on NCBI reference sequence build 36 and dbSNP build 126. **B**: Red line indicates the recombination rate (cM/Mb) estimated from a HapMap JPT (release 28) dataset. Purple line indicates cumulative genetic distance (in cM) from the strongest signal. Signal boundaries (vertical dotted lines) were chosen using the recombination hot spots flanking the strongest signal. **C**: Blue lines (intron) and boxes (exon) indicate the locations of known genes. **D**: Linkage disequilibrium (LD) map calculated using the Hapmap JPT dataset.

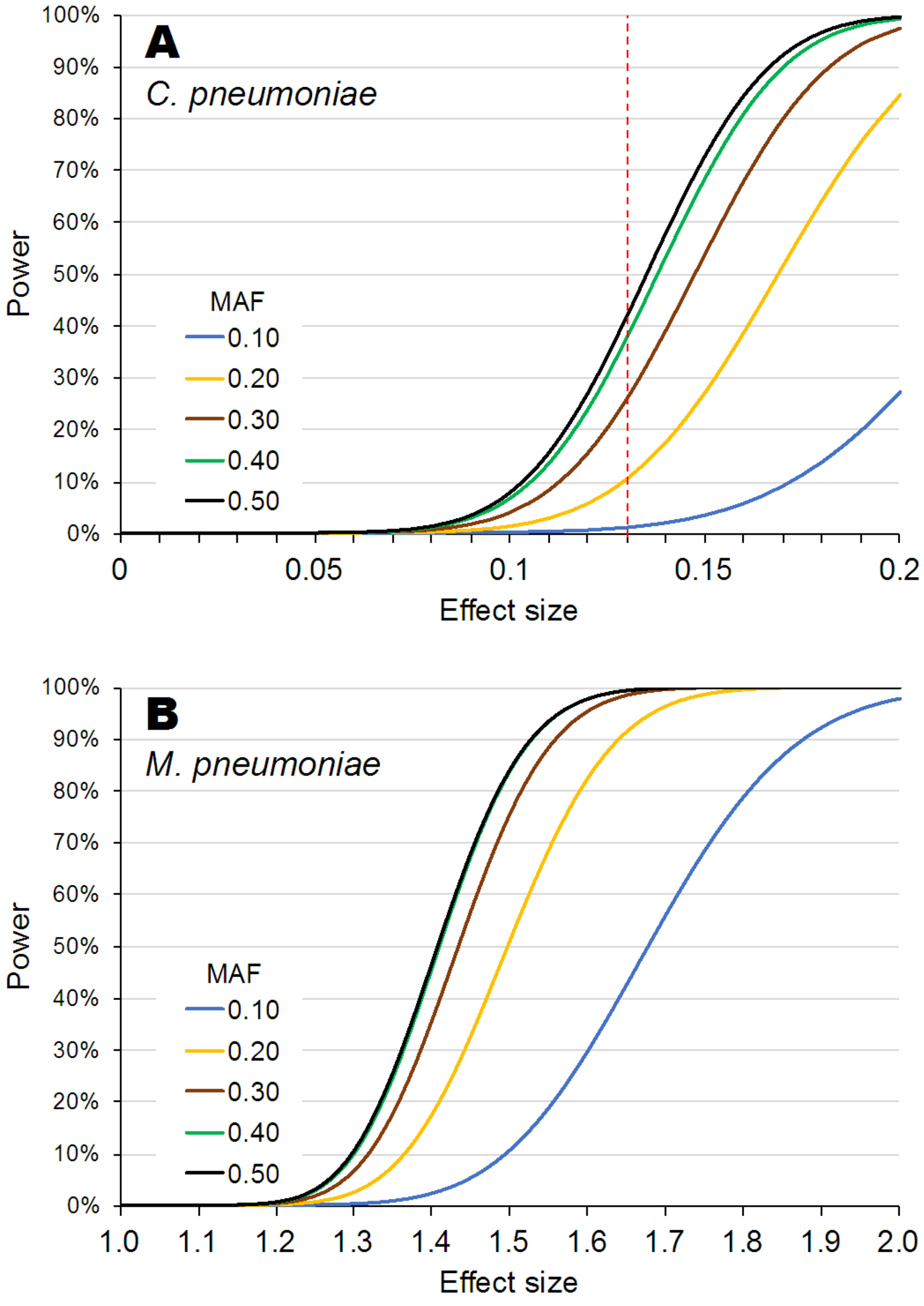

Supplementary Figure 8. Statistical power of the present GWAS

Statistical power of the present GWAS of *C. pneumoniae* IgA and IgG indices (mean=0, standard deviation=1) and *M. pneumoniae* seropositivity (case:unmatched control=1:3.9) was calculated under additive genetic model using Quanto software[12, 13]. Red dotted line indicates presently found rs17634369 SNP.

Supplementary Table 1 Clinical characteristics of study subjects

	GWAS (n=3,246)	Replication (n=5,991)
Age (years)	52±14	54±13
Sex (male/female)	1,092/2,154	1,930/4,061
Body mass index (kg/m^2)	22.3±3.1	22.3±3.3
Smoking (current/past/never)	540/654/2,052	795/1,236/3,960
C. pneumoniae IgA index	1.00±0.64	0.99±0.64
C. pneumoniae IgG index	1.13±0.71	1.23±0.76

Among a total of 9,804 subjects, genome-wide SNP genotype data was available for 3,710 subjects. Samples of participants meeting any of the following conditions were excluded from the analysis; a call rate less than 95% (n=162), showed high degrees of kinship (n=295), and ancestry outliers (n=7). Among a remaining samples, a total of 5,991 samples were available for replication analysis.

Supplementary Table 2. Differences in clinical parameters by severity of pulmonary function.

		FEV_1/FVC (%)				*P*
		≥80%	≥70%	≥60%	<60%	
Male (n=2,953)	n	1,587	1,158	171	37	
	Frequency of COPD (%)	93.0		7.0		
	Age (years)	52±14	59±12	63±9	65±9	<0.001
	Body mass index (kg/m^2)	23.7±3.2	23.1±2.7	22.7±2.7	21.6±2.9	<0.001
	Smoking (current or past, %)	71.5	78.7	81.3	94.6	<0.001
	Medication for COPD (%)	0.1	0.2	1.2	18.9	<0.001
	Medication for asthma (%)	0.4	0.8	2.9	2.7	0.199
Female (n=6,087)	n	4,623	1,331	122	11	
	Frequency of COPD (%)	97.8		2.2		
	Age (years)	52±13	56±12	55±14	61±13	<0.001
	Body mass index (kg/m^2)	21.9±3.4	21.3±2.8	20.8±2.4	21.3±3.3	<0.001
	Smoking (current or past, %)	15.4	13.9	18.9	27.3	0.227
	Medication for COPD (%)	0.1	0.3	0.8	0	0.031
	Medication for asthma (%)	0.5	1.2	7.4	0	0.232

Values are mean ± standard deviation.

FEV_1, forced expiratory volume in 1 sec; FVC, forced vital capacity; COPD, chronic obstructive pulmonary disease. COPD was defined as a ratio of FEV1 to FVC of less than 70%.

Differences in clinical parameters were assessed by analysis of variance, while differences in frequencies were evaluated by a chi-square test.

Supplementary Table 3. Differences in clinical parameters by *C. pneumoniae* and *M. pneumoniae* seropositivity (n=8,950)

	C. pneumoniae				*M. pneumoniae*			
	Seronegative (6,623)	Seropositive (2,327)	*P*		Seronegative (7,128)	Seropositive (1,822)	*P*	
			Crude	Adjusted			Crude	Adjusted
FVC (L)	3.26±0.84	3.23±0.80	0.057	<0.001	3.24±0.84	3.32±0.80	<0.001	0.251
FVC % predicted (%)	105±15	103±15	<0.001	0.001	105±15	104±15	0.481	0.493
FEV_1 (L)	2.69±0.68	2.62±0.65	<0.001	<0.001	2.65±0.67	2.76±0.65	<0.001	0.033
FEV_1/FVC (%)	82.7±6.3	81.4±6.9	<0.001	<0.001	82.1±6.3	83.4±7.0	<0.001	0.164
FEV_1 % predicted (%)	104±16	102±17	<0.001	0.003	104±16	103±16	0.087	0.149
COPD (%)	2.9	5.5	<0.001	0.003	3.6	3.6	0.904	0.003

Subjects under treatment for asthma or chronic obstructive pulmonary disease (COPD) (n=90) were excluded from analysis.

Values are mean ± standard deviation.

Seropositivity for *C. pneumoniae* was defined as both IgA and IgG index values of more than 1.1. Titers of antibody to *M. pneumoniae* greater than 1:40 were considered as seropositive. COPD was defined as a ratio of FEV1 to FVC of less than 70%.

Differences in clinical parameters were assessed by analysis of variance, while differences in frequencies were evaluated by a chi-square test. *P*-values adjusted for age, sex, body height, body weight, and Brinkman index were calculated by multiple regression analyses.

Supplementary Table 4. Top-hit SNPs in GWAS of *C. pneumoniae* IgA index value

Chr	rs Number	GWAS				Replication			Combined		
		Coded allele (freq)	n (HWE p)	Beta (s.e.)	*P*	n (HWE p)	Beta (s.e.)	*P*	n (HWE p)	Beta (s.e.)	*P*
6	rs9460391	T (0.196)	3,246 (0.857)	0.163 (0.030)	$6.90^{*}10^{-8}$	5,885 (0.506)	0.018 (0.023)	0.414			
7	rs17634369	G (0.558)	3,246 (0.713)	0.134 (0.024)	$2.96^{*}10^{-8}$	5,900 (0.543)	0.080 (0.018)	$1.36^{*}10^{-5}$	9,146 (0.479)	0.099 (0.015)	$1.32^{*}10^{-11}$
13	rs942102	C (0.342)	3,246 (0.117)	-0.134 (0.025)	$7.73^{*}10^{-8}$	5,928 (0.960)	0.010 (0.019)	0.621			

C. pneumoniae IgA index value was transformed by rank-based inverse normal transformation. Beta and *P*-values in the additive regression models adjusted for age, age squared, sex, and BMI are shown. Combined analysis was performed using individual data. HWE, Hardy-Weinberg equilibrium; s.e., standard error.

Supplementary Table 5. Associations between known genotypes susceptible for systemic lupus erythematosus and *C. pneumoniae* IgA index values

SNPs for SLE			*C. pneumoniae* IgA index value		
Locus	Gene	rs No.	beta	Se	*P*
1q25	TNFSF4	rs2205960	-0.001	0.031	0.967
1q31	IL10	rs3024505	0.162	0.094	0.084
2p13	TET3	rs6705628	0.004	0.031	0.893
2p22	RASGRP3	rs13385731	0.056	0.034	0.099
2q32	STAT4	rs7574865	0.036	0.025	0.148
3p14	PXK	rs6445975	-0.044	0.029	0.128
3q13	TMEM39A-CD80	rs6804441	-0.013	0.026	0.615
4q21	AFF1	rs340630	0.026	0.024	0.296
4q24	BANK1	rs10516487	-0.005	0.041	0.909
5q33	TNIP1	rs10036748	0.025	0.027	0.367
6p21	UHRF1BP1	rs11755393	-0.020	0.026	0.452
6p21	HLA region	rs9501626	-0.048	0.038	0.212
6q21	PRDM1-ATG5	rs548234	0.015	0.026	0.556
6q23	TNFAIP3	rs2230926	-0.142	0.047	0.003
7q11	HIP1	rs6964720	-0.026	0.029	0.378
7q32	IRF5	rs4728142	0.001	0.039	0.972
8p23	BLK	rs2254546	-0.003	0.026	0.916
10q11	LRRC18-WDFY4	rs1913517	0.028	0.027	0.305
10q21	ARID5B	rs4948496	-0.014	0.025	0.557
11p13	CD44	rs2732552	-0.027	0.027	0.315
11p15	PHRF1	rs4963128	0.002	0.063	0.976
11q23	CXCR5	rs10892301	0.002	0.025	0.950
11q23	ETS1	rs1128334	0.065	0.025	0.010
11q23	PHLDB1	rs11603023	0.026	0.028	0.362
12p13	CREBL2-CDKN1B	rs10845606	0.004	0.029	0.902
12q23	DRAM1	rs4622329	0.017	0.024	0.475
12q24	SLC15A4	rs1385374	0.012	0.032	0.716
16p11	ITGAM	rs7197475	-0.059	0.040	0.141
16q24	IRF8	rs11644034	0.083	0.037	0.025
22q11	HIC2-UBE2L3	rs463426	0.010	0.024	0.672

C. pneumoniae IgA index values were transformed by rank-based inverse normal transformation, and association analysis in the GWAS sample (Supplemental Table 1) was performed by linear regression analysis under additive genetic model adjusted for age, age squared, sex, and BMI (PLINK, v1.07)[1].

Genetic loci that have been validated to be associated with systemic lupus erythematosus (SLE) ($p<5.0^{*}10^{-8}$) by previous genome-wide association studies[2-10] were selected.

Supplementary Table 6. Multiple logistic regression analysis for COPD (n=8,950)

	Odds (95% C.I.)	*P*
Age (10 years)	1.67 (1.48-1.89)	<0.001
Sex (male)	1.16 (0.77-1.73)	0.479
Body height (cm)	1.09 (1.06-1.11)	<0.001
Body weight (kg)	0.95 (0.94-0.97)	<0.001
Brinkman index (100 index)	1.10 (1.07-1.13)	<0.001
C. pneumoniae seropositive	1.41 (1.11-1.80)	0.005
M. pneumoniae seropositive	1.54 (1.14-2.07)	0.005
rs17634369 genotype (per allele)	1.11 (0.94-1.31)	0.227

Subjects under treatment for asthma or chronic obstructive pulmonary disease (COPD) (n=90) were excluded from analysis.

C.I., confidence interval.

REFERENCES

1. Purcell S, Neale B, Todd-Brown K, Thomas L, Ferreira MA, Bender D, Maller J, Sklar P, de Bakker PI, Daly MJ, Sham PC. PLINK: a tool set for whole-genome association and population-based linkage analyses. ***Am J Hum Genet.*** 2007;**81**:559-575.
2. Kozyrev SV, Abelson AK, Wojcik J, Zaghlool A, Linga Reddy MV, Sanchez E, Gunnarsson I, Svenungsson E, Sturfelt G, Jönsen A, Truedsson L, Pons-Estel BA, Witte T, D'Alfonso S, Barizzone N, Danieli MG, Gutierrez C, Suarez A, Junker P, Laustrup H, González-Escribano MF, Martin J, Abderrahim H, Alarcón-Riquelme ME. Functional variants in the B-cell gene BANK1 are associated with systemic lupus erythematosus. ***Nat Genet***. 2008;**40**:211-216.
3. Han JW, Zheng HF, Cui Y, Sun LD, Ye DQ, Hu Z, Xu JH, Cai ZM, Huang W, Zhao GP, Xie HF, Fang H, Lu QJ, Xu JH, Li XP, Pan YF, Deng DQ, Zeng FQ, Ye ZZ, Zhang XY, Wang QW, Hao F, Ma L, Zuo XB, Zhou FS, Du WH, Cheng YL, Yang JQ, Shen SK, Li J, Sheng YJ, Zuo XX, Zhu WF, Gao F, Zhang PL, Guo Q, Li B, Gao M, Xiao FL, Quan C, Zhang C, Zhang Z, Zhu KJ, Li Y, Hu DY, Lu WS, Huang JL, Liu SX, Li H, Ren YQ, Wang ZX, Yang CJ, Wang PG, Zhou WM, Lv YM, Zhang AP, Zhang SQ, Lin D, Li Y, Low HQ, Shen M, Zhai ZF, Wang Y, Zhang FY, Yang S, Liu JJ, Zhang XJ. Genome-wide association study in a Chinese Han population identifies nine new susceptibility loci for systemic lupus erythematosus. ***Nat Genet.*** 2009;**41**:1234-1237.
4. Gateva V, Sandling JK, Hom G, Taylor KE, Chung SA, Sun X, Ortmann W, Kosoy R, Ferreira RC, Nordmark G, Gunnarsson I, Svenungsson E, Padyukov L, Sturfelt G, Jönsen A, Bengtsson AA, Rantapää-Dahlqvist S, Baechler EC, Brown EE, Alarcón GS, Edberg JC, Ramsey-Goldman R, McGwin G Jr, Reveille JD, Vilá LM, Kimberly RP, Manzi S, Petri MA, Lee A, Gregersen PK, Seldin MF, Rönnblom L, Criswell LA, Syvänen AC, Behrens TW, Graham RR. A large-scale replication study identifies TNIP1, PRDM1, JAZF1, UHRF1BP1 and IL10 as risk loci for systemic lupus erythematosus. ***Nat Genet.***

2009;**41**:1228-1233.

5. Yang W, Shen N, Ye DQ, Liu Q, Zhang Y, Qian XX, Hirankarn N, Ying D, Pan HF, Mok CC, Chan TM, Wong RW, Lee KW, Mok MY, Wong SN, Leung AM, Li XP, Avihingsanon Y, Wong CM, Lee TL, Ho MH, Lee PP, Chang YK, Li PH, Li RJ, Zhang L, Wong WH, Ng IO, Lau CS, Sham PC, Lau YL; Asian Lupus Genetics Consortium. Genome-wide association study in Asian populations identifies variants in ETS1 and WDFY4 associated with systemic lupus erythematosus. ***PLoS Genet.*** 2010;**6**:e1000841
6. Lessard CJ, Adrianto I, Kelly JA, Kaufman KM, Grundahl KM, Adler A, Williams AH, Gallant CJ; Marta E. Alarcón-Riquelme on behalf of the BIOLUPUS and GENLES Networks, Anaya JM, Bae SC, Boackle SA, Brown EE, Chang DM, Criswell LA, Edberg JC, Freedman BI, Gregersen PK, Gilkeson GS, Jacob CO, James JA, Kamen DL, Kimberly RP, Martin J, Merrill JT, Niewold TB, Park SY, Petri MA, Pons-Estel BA, Ramsey-Goldman R, Reveille JD, Song YW, Stevens AM, Tsao BP, Vila LM, Vyse TJ, Yu CY, Guthridge JM, Bruner GR, Langefeld CD, Montgomery C, Harley JB, Scofield RH, Gaffney PM, Moser KL. Identification of a systemic lupus erythematosus susceptibility locus at 11p13 between PDHX and CD44 in a multiethnic study. ***Am J Hum Genet.*** 2011;**88**:83-91.
7. Okada Y, Shimane K, Kochi Y, Tahira T, Suzuki A, Higasa K, Takahashi A, Horita T, Atsumi T, Ishii T, Okamoto A, Fujio K, Hirakata M, Amano H, Kondo Y, Ito S, Takada K, Mimori A, Saito K, Kamachi M, Kawaguchi Y, Ikari K, Mohammed OW, Matsuda K, Terao C, Ohmura K, Myouzen K, Hosono N, Tsunoda T, Nishimoto N, Mimori T, Matsuda F, Tanaka Y, Sumida T, Yamanaka H, Takasaki Y, Koike T, Horiuchi T, Hayashi K, Kubo M, Kamatani N, Yamada R, Nakamura Y, Yamamoto K. A genome-wide association study identified AFF1 as a susceptibility locus for systemic lupus eyrthematosus in Japanese. ***PLoS Genet.*** 2012;**8**:e1002455.
8. Lessard CJ, Adrianto I, Ice JA, Wiley GB, Kelly JA, Glenn SB, Adler AJ, Li H,

Rasmussen A, Williams AH, Ziegler J, Comeau ME, Marion M, Wakeland BE, Liang C, Ramos PS, Grundahl KM, Gallant CJ, Alarcón-Riquelme ME, Alarcón GS, Anaya JM, Bae SwC, Boackle SA, Brown EE, Chang DM, Cho SK, Criswell LA, Edberg JC, Freedman BI, Gilkeson GS, Jacob CO, James JA, Kamen DL, Kimberly RP, Kim JH, Martin J, Merrill JT, Niewold TB, Park SY, Petri MA, Pons-Estel BA, Ramsey-Goldman R, Reveille JD, Scofield RH, Song YW, Stevens AM, Tsao BP, Vila LM, Vyse TJ, Yu CY, Guthridge JM, Kaufman KM, Harley JB, Wakeland EK, Langefeld CD, Gaffney PM, Montgomery CG, Moser KL; BIOLUPUS Network; GENLES Network. Identification of IRF8, TMEM39A, and IKZF3-ZPBP2 as susceptibility loci for systemic lupus erythematosus in a large-scale multiracial replication study. ***Am J Hum Genet.*** 2012;**90**:648-660.

9. Yang W, Tang H, Zhang Y, Tang X, Zhang J, Sun L, Yang J, Cui Y, Zhang L, Hirankarn N, Cheng H, Pan HF, Gao J, Lee TL, Sheng Y, Lau CS, Li Y, Chan TM, Yin X, Ying D, Lu Q, Leung AM, Zuo X, Chen X, Tong KL, Zhou F, Diao Q, Tse NK, Xie H, Mok CC, Hao F, Wong SN, Shi B, Lee KW, Hui Y, Ho MH, Liang B, Lee PP, Cui H, Guo Q, Chung BH, Pu X, Liu Q, Zhang X, Zhang C, Chong CY, Fang H, Wong RW, Sun Y, Mok MY, Li XP, Avihingsanon Y, Zhai Z, Rianthavorn P, Deekajorndej T, Suphapeetiporn K, Gao F, Shotelersuk V, Kang X, Ying SK, Zhang L, Wong WH, Zhu D, Fung SK, Zeng F, Lai WM, Wong CM, Ng IO, Garcia-Barceló MM, Cherny SS, Shen N, Tam PK, Sham PC, Ye DQ, Yang S, Zhang X, Lau YL. Meta-analysis followed by replication identifies loci in or near CDKN1B, TET3, CD80, DRAM1, and ARID5B as associated with systemic lupus erythematosus in Asians. ***Am J Hum Genet***. 2013;**92**:41-51.

10. Zhang J, Zhang Y, Yang J, Zhang L, Sun L, Pan HF, Hirankarn N, Ying D, Zeng S, Lee TL, Lau CS, Chan TM, Leung AM, Mok CC, Wong SN, Lee KW, Ho MH, Lee PP, Chung BH, Chong CY, Wong RW, Mok MY, Wong WH, Tong KL, Tse NK, Li XP, Avihingsanon Y, Rianthavorn P, Deekajorndej T, Suphapeetiporn K, Shotelersuk V, Ying SK, Fung SK, Lai

WM, Garcia-Barceló MM, Cherny SS, Tam PK, Cui Y, Sham PC, Yang S, Ye DQ, Zhang XJ, Lau YL, Yang W. Three SNPs in chromosome 11q23.3 are independently associated with systemic lupus erythematosus in Asians. ***Hum Mol Genet.*** 2013 in press.

11. Price AL, Patterson NJ, Plenge RM, Weinblatt ME, Shadick NA, Reich D. Principal components analysis corrects for stratification in genome-wide association studies. ***Nat Genet***. 2006;**38**:904-909.
12. Gauderman WJ. Sample size requirements for association studies of gene-gene interaction. ***Am J Epidemiol.*** 2002;155:478-484.
13. Gauderman WJ. Sample size requirements for matched case-control studies of gene-environment interaction. ***Stat Med.*** 2002;21:35-50.
